# Fundamental Role of Pentose Phosphate Pathway within the Endoplasmic Reticulum in Glutamine Addiction of Triple-Negative Breast Cancer Cells

**DOI:** 10.3390/antiox12010043

**Published:** 2022-12-26

**Authors:** Cecilia Marini, Vanessa Cossu, Sonia Carta, Elisa Greotti, Daniela Gaglio, Nadia Bertola, Sabrina Chiesa, Silvia Bruno, Francesca Vitale, Marcella Bonanomi, Danilo Porro, Mattia Riondato, Anna Maria Orengo, Matteo Bauckneht, Silvia Morbelli, Silvia Ravera, Gianmario Sambuceti

**Affiliations:** 1 Institute of Molecular Bioimaging and Physiology (IBFM), National Research Council (CNR), 20054 Milan, Italy; 2 IRCCS Ospedale Policlinico San Martino, 16132 Genova, Italy; 3 Department of Biomedical Sciences, University of Padova, 35131 Padua, Italy; 4 Neuroscience Institute, Italian National Research Council (CNR), 35131 Padua, Italy; 5 Padova Neuroscience Center (PNC), University of Padova, 35131 Padua, Italy; 6 ISBE. IT, Centre of Systems Biology, 20126 Milan, Italy; 7 Elixir Infrastructure and NBFC, National Biodiversity Future Center, 90133 Palermo, Italy; 8 Department of Experimental Medicine, Human Anatomy, University of Genoa, 16132 Genova, Italy; 9 Department of Biotechnology and Biosciences, University of Milano-Bicocca, Piazza della Scienza 2, 20126 Milan, Italy; 10 Department of Health Sciences, University of Genoa, 16132 Genova, Italy

**Keywords:** glutamine metabolism, breast cancer, pentose phosphate pathway, redox balance, hexose-6-phosphate-dehydrogenase, ^18^F-fluoro-deoxy-glucose, endoplasmic reticulum, endoplasmic reticulum–mitochondria network, calcium flux

## Abstract

Cancer utilization of large glutamine equivalents contributes to diverging glucose-6-P flux toward the pentose phosphate shunt (PPP) to feed the building blocks and the antioxidant responses of rapidly proliferating cells. In addition to the well-acknowledged cytosolic pathway, cancer cells also run a largely independent PPP, triggered by hexose-6P-dehydrogenase within the endoplasmic reticulum (ER), whose activity is mandatory for the integrity of ER–mitochondria networking. To verify whether this reticular metabolism is dependent on glutamine levels, we complemented the metabolomic characterization of intermediates of the glucose metabolism and tricarboxylic acid cycle with the estimation of proliferating activity, energy metabolism, redox damage, and mitochondrial function in two breast cancer cell lines. ER-PPP activity and its determinants were estimated by the ER accumulation of glucose analogs. Glutamine shortage decreased the proliferation rate despite increased ATP and NADH levels. It depleted NADPH reductive power and increased malondialdehyde content despite a marked increase in glucose-6P-dehydrogenase. This paradox was explained by the deceleration of ER-PPP favored by the decrease in hexose-6P-dehydrogenase expression coupled with the opposite response of its competitor enzyme glucose-6P-phosphatase. The decreased ER-PPP activity eventually hampered mitochondrial function and calcium exchanges. These data configure the ER-PPP as a powerful, unrecognized regulator of cancer cell metabolism and proliferation.

## 1. Introduction

In addition to the accelerated glycolytic flux described by Warburg almost a century ago [1,2,3,4,5], a cancer cell utilizes large amounts of glutamine to balance the high need for energy and building blocks asked by its reductive biosynthesis despite harsh microenvironmental conditions [6]. The high proliferation rate, indeed, implies a large use of glutamine g-nitrogen to synthesize nucleotides and non-essential amino acids [7,8,9]. Likewise, cancer growth also implies the high-rate synthesis of fatty acids and thus a high-rate of acetyl-CoA generation warranted by the citrate export from the tricarboxylic acid cycle (TCA) to the cytosol [10]. Carboxylation of pyruvate to oxaloacetate is inhibited in the mitochondria of many cancers, raising the need to compensate for the high efflux of carbon equivalents by enhancing the influx of glutamine-derived glutamate and its conversion to alpha-ketoglutarate [11].

In addition to its interference with mitochondrial function, glutamine can also regulate glucose metabolism in the cytosol [12]. Indeed, its processing by glutamine-fructose-6-phosphate transaminase fuels the generation of uridine-diphosphate-N-acetyl glucosamine (UDP-N-Ac-Gla) that diverges glucose flux to the pentose phosphate pathway [PPP] by inhibiting the catalytic function of the glycolysis rate-limiting enzyme phospho-fructo-kinase [12]. This pathway would thus configure glutamine as a primary regulator of PPP activity in cancer cells. In agreement with this observation, amino acid withdrawal has been shown to induce evident redox damage and to decrease the proliferation rate of cancer cells despite their increased expression of the PPP-triggering enzyme glucose-6P-dehydrogenase (G6PD) [13].

In addition to its role in fueling the ribose-5-phosphate for nucleic acid synthesis, PPP represents the main provider of NADPH-reductive power for antioxidant responses and the synthesis of fatty acids needed by the membrane generation of a rapidly growing tumor mass. In the present study, we thus verified whether, through which pathways, and to what degree, glutamine regulates glucose-6-phophate (G6P) flux through the PPP. For this purpose, we extended our analysis beyond the well-recognized G6PD-triggered metabolism to consider its largely autonomous and apparently independent counterpart, triggered by hexose-6-phosphate dehydrogenase (H6PD) and confined within the endoplasmic reticulum (ER) [14,15]. Indeed, H6PD expression and catalytic function have been found to play a mandatory for the high proliferation rate and migratory potential of breast cancer cells [16], in agreement with its ER confinement that configures this enzyme as a fundamental regulator of ER–mitochondria networking [17,18] and Ca^2+^ exchanges [16].

Evaluating the relative contribution of cytosolic and ER-PPPs, here we show that the decelerated proliferation rate induced by glutamine shortage is associated with selective impairment of ER-PPP, eventually resulting in mitochondrial dysfunction due to significant alteration of the ER–mitochondria contact points.

## 2. Materials and Methods

### 2.1. Cell Culture

MCF7 and MDA-MB-231 (MDA) cells were cultured in Dulbecco’s Modified Eagle Medium (DMEM, Gibco, Los Angeles, CA, USA) supplemented with 10% fetal bovine serum, 25 mM glucose, 2 mM glutamine, 1 mM pyruvate, 1% penicillin-streptomycin (Gibco, Grand Island, NY, USA) at 37 °C with 5% CO_2_.

For each experiment, cells were seeded using a control DMEM medium. Five hours after cell seeding, when cells became adherent, the cell cultures were exposed to either fresh control medium (CTR) or DMEM containing only 0.5 mM glutamine (plus all other above supplementations) (LG). Cells were harvested for measurements 48 h later.

### 2.2. Cell Viability and Proliferation Assays

Cell growth of the tumor cell line cultures was assessed by counting trypan blue-negative cells.

Cell viability was evaluated by propidium iodide (PI) exclusion assays. Cells were stained with 1 μg/mL PI (Enzo Life Sciences, Executive Blvd, Farmingdale, NY 11735, USA) and PI fluorescence was measured using a FACScan Flow Cytometer (Becton Dickinson, Milan, Italy).

Cell proliferation rate was evaluated using carboxyfluorescein diacetate succinimidyl ester (CFSE) (by Sigma incorporated in Merck KGaA, Frankfurter Str. 250, 64293 Darmstadt, Germany). Briefly, cells were plated in 6-well plates and 24 h later adherent cells were labeled in prewarmed phosphate-buffered saline (PBS) containing CFSE at a concentration of 0.3 μM. Cells were incubated for 20 min at 37 °C. After replacing the labeling solution with a fresh prewarmed cell culture medium, cells were incubated for another 20 min at 37 °C to remove unincorporated CFSE. The cells were then cultured either with fresh standard DMEM medium (CTR) or low medium LG. After 24 and 48 h, cells were detached from the substrate by trypsinization, and CFSE fluorescence was acquired on a FACSCan flow cytometer. Flow cytometry data were analyzed by FlowJo software (Version 8.7, Becton–Dickinson, Franklin Lakes, NJ, USA).

### 2.3. Seahorse Analysis

MDA and MCF7 oxygen consumption rate (OCR) and extracellular acidification rate (ECAR) were determined using a Seahorse XFp Extracellular Flux Analyzer (Agilent Technologies, Santa Clara, CA, USA). A quantity of 4000 or 7000 cells/well (for MDA or MCF7, respectively) were seeded in XFp plates and incubated in a control medium. After 5 h, the medium was replaced, and cells were incubated with either control or LG medium. After 48 h, OCR and ECAR were monitored according to the manufacturer’s instructions. Briefly, the incubation medium was replaced with Agilent Seahorse DMEM, pH 7.4, enriched with nutrients mimicking the respective incubation medium conditions. Three measurements of OCR and ECAR were taken for the baseline and after sequential injection of the selective glutaminase inhibitor Bis-2-(5-phenylacetamido-1,3,4-thiadiazol-2-yl)ethyl sulfide (BPTES, 3 µM), the ATP-synthase inhibitor Oligomycin A (1.5 µM), the ATP synthesis uncoupler carbonyl cyanide-4-trifluoromethoxyphenylhydrazone (FCCP, 1.25 µM), and the Complex I + Complex III inhibitors (rotenone + antimycin A, 0.5 µM).

OCR was expressed as picomol O_2_ × min^−1^/million cells of cells while ECAR was converted to proton efflux rate (PER) using WAVE software (Version 2.4, Agilent) after evaluating the buffer factor of the control and LG medium. To calculate the nanomoles of glucose used through glycolysis, PER value expressed in picomol H^+^ × min^−1^/million cells of cells was converted to glucose nanomoles × min^−1^/million cells, applying the equation Glucose + 2 ADP + 2 Pi --> 2 Lactate + 2 ATP + 2 H_2_O + 2 H^+^.

### 2.4. Metabolites Extraction

Cells, seeded in 6-well plates, were quickly rinsed with NaCl 0.9% and quenched with 500 μL ice-cold 70:30 acetonitrile:water. Plates were placed at −80 °C for 10 min, then cells were collected by scraping and sonicated 5 s for 5 pulses at 70% power twice. Samples were centrifuged at 12,000× *g* for 10 min and supernatants were collected in a glass insert and dried in a centrifugal vacuum concentrator (Concentrator plus/Vacufuge plus, Eppendorf) at 30 °C for about 2.5 h. Samples were then resuspended with 150 μL H_2_O before analyses.

### 2.5. Liquid Chromatography with Tandem Mass Spectrometry Metabolic Profiling

Liquid chromatography with tandem mass spectrometry (LC-MS) was performed using an Agilent 1290 Infinity ultrahigh performance LC (UHPLC) system and an InfintyLab Poroshell 120 PFP column (2.1  ×  100 mm, 2.7 μm; Agilent Technologies) coupled with a quadrupole time-of-flight hybrid mass spectrometer (Agilent 6550 iFunnel Q-TOF) and equipped with an electrospray Dual JetStream source operated in negative mode.

The injection volume was 15 μL, and the flow rate was 0.2 mL/min with column temperature set at 35 °C. Both mobile phases A (100% water) and B (100% acetonitrile) contained 0.1% formic acid, the injection volume was 15 μL, and LC gradient conditions were: 0 min: 100% A; 2 min: 100% A; 4 min: 99% A; 10 min: 98% A;11 min: 70% A; 15 min: 70% A; 16 min: 100% A with 5 min of post-run. The flow rate was 0.2 mL/min and the column temperature was 35 °C.

Mass spectra were recorded in centroid mode in a mass range from *m/z* 60 to 1050 *m*/*z*.

The mass spectrometer operated using a capillary voltage of 3.7 kV. The source temperature was set to 285 °C, with 14 L/min drying gas and a nebulizer pressure of 45 psig. Fragmentor, skimmer, and octopole voltages were set to 175, 65, and 750 V, respectively.

Active reference mass correction was performed through a second nebulizer using the reference solution (*m/z* 112.9855 and 1033.9881) dissolved in mobile phase 2-propanol–acetonitrile–water (70:20:10 *v/v*). Data were acquired from *m/z* 60–1050. Data analysis and isotopic natural abundance correction were performed with MassHunter ProFinder (Agilent). Normalization was performed based on protein content.

### 2.6. NADP/NADPH Ratio and Malondialdehyde Assay

NADP/NADPH ratio was tested using a dedicated Assay Kit (Abcam; Cat#ab65349), following the manufacturer’s instructions. Malondialdehyde (MDA) levels were evaluated by the thiobarbituric acid-reactive substance assay [18,19]. In all cases, enzymatic activity was normalized for total protein concentrations tested using Bradford analysis [20].

### 2.7. Radioactive Glucose Analog Uptake and its Relationship with Glycolytic Flux

In vitro ^18^F-Fluoro-deoxyglucose (FDG) uptake was estimated using the LigandTracer White^®^ instrument (Ridgeview, Uppsala, SE) according to our previously validated procedure [21,22,23]. Briefly, the device consists of a beta-emission detector and a rotating platform harboring a standard Petri dish. The rotation axis is inclined at 30° from the vertical so that the organ alternates its position from the nadir (for incubation) to the zenith (for counting) every minute.

To calibrate the measured counting rate, a square of absorbent paper (5 mm × 5 mm) was stuck on the counted Petri point and soaked with FDG (200 to 600 KBq). Radioactivity decay was thus monitored for 12 h as shown in Supplementary Material Figure S1. The calibration factor was checked every week and was set by the slope of the regression line to 0.03 ± 0.0006. According to this process, the measured counting rate (in counts per second or cps) was thus divided by 0.03 to define the true radioactivity content in the tested sample.

The same culture used for the Seahorse analysis was used to collect 10–15 × 10^3^ cells of each type that were seeded and cultured for two days. Soon before the experiment, cells were immersed in 3 mL of the same culture medium containing 1.8 to 2.2 MBq/mL FDG. After 120 min monitoring, obtained counting rate was normalized according to the following equation:

(1) $$FDG\;culture\;content=\frac{Culture\;counting\;rate\;\left(cps\right)}{calibration\;factor\times administered\;radioactivity\;\left(Bq\right)\times cell\;number}$$

The conventional Sokoloff model of FDG uptake [24], was used to estimate the metabolic rate of glucose (MRGlu*) as the product of retained FDG fraction times the total available glucose (5.5 × 3 = 15 µMol) eventually divided for the observation period of 120 m.

This estimation was thus divided by the direct measurement of glycolytic-related glucose disposal (MRGlu) provided by the Seahorse approach in the corresponding experimental condition and on the same day. The ratio MRGlu*/MRGlu also called lumped constant [24], was thus calculated.

### 2.8. Western Blot Analysis

Protein extraction was performed in RIPA buffer supplemented with a protease inhibitor cocktail. Western blot (WB) experiments were performed according to the standard procedure with the following antibodies: anti-hexose 6-phosphate dehydrogenase (H6PD, #ab170895, Abcam, Cambridge, UK), anti-glucose 6-phosphate dehydrogenase (G6PD, #ab124738, Abcam, Cambridge, UK), anti-glucose 6-phosphate transport (G6PT or SLC37A4, #ab80463, Abcam, Cambridge, UK), anti-glucose-6-phosphatase (G6Pase catalytic subunit, #ab83690, Abcam, Cambridge, UK), anti-mitofusin 2 (MFN2, #PA5-72811, ThermoFisher Scientific, Waltham, MA, USA), anti-dynamin-related protein 1 (#DRP1-101AP, ThermoFisher Scientific, Waltham, MA, USA), anti-translocase of the inner membrane (TIM) (TIM subunit 17A, #ab192246, Abcam, Cambridge, UK), anti-mitochondrial calcium uniporter (MCU, HPA016480 Sigma-Aldrich, and anti-β-actin (#15G5A11/E2, ThermoFisher Scientific, Waltham, MA, USA) or anti-HSP90 (BD Bioscience, BD610418 as the loading control. Specific secondary antibodies were employed (Sigma-Aldrich, St. Louis, MO, USA), all diluted 1:10,000 in PBS-Tween. Bands were detected and analyzed for optical density using an enhanced chemiluminescence substrate (ECL, Bio-Rad, Hercules, CA, USA), a chemiluminescence system (Alliance 6.7 WL 20M, UVITEC, Cambridge, UK), and UV1D software (UVITEC, Cambridge, UK). All the bands of interest were normalized with actin levels detected on the same membrane.

### 2.9. 2-NBDG/ER Colocalization

2-[N-(7-nitrobenz-2-oxa-1,3-diazol-4-yl)amino]-2-deoxyglucose (2-NBDG) colocalization with the ER was performed by confocal microscopy as previously described [25]. Briefly, cells grown on 12 mm diameter glass coverslips were stained for 15 min with the fluorescent probes 2-NBDG (25 microM) and glibenclamide (0.5 mM) at 37 °C. After extensive washing, the cells were immediately imaged with an SP2-AOBS confocal microscope (Leica Microsystems, Mannheim, Germany). Four to eight randomly selected fields were analyzed in three independent samples for each treatment. Original unadjusted and uncorrected images were processed by Fiji software (Version 2.3, NIH). Colocalization was expressed as the percent of 2-NBDG-containing pixels that colocalize with glibenclamide-positive ones and glibenclamide-containing pixels that colocalize with 2-NBDG-positive ones.

### 2.10. Mitochondrial–ER Colocalization and Mitochondrial Morphology

Cells were seeded onto glass coverslips (18 mm diameter for imaging experiments) and transfection was performed at 60% confluence using Lipofectamine 2000 (Invitrogen) with 1 µg of DNA. Split-GFP-based contact site sensors (SPLICSs) were used as previously described [26,27]. Images were collected on a Leica TCS SP5 II confocal system using a WLL white laser (Leica) equipped with a PlanApo 100× (numerical aperture 1.4 objective). The pinhole was set to 1 Airy unit and the imaging was performed at 1024 × 1024 pixels per image, with a 0.2 Hz acquisition rate. Photomultiplier gain was adjusted and maintained among different experiments to minimize background and avoid saturation. Images were elaborated with Fiji software (Version 2.3, NIH). Mitochondrial–ER colocalization: To count ER–mitochondria contacts, a complete z-stack of the cell was acquired every 0.42 µm. Z-stacks were processed using Fiji. Images were first convolved and filtered using the Gaussian blur filter. A 3D reconstruction of the resulting image was obtained using the Volume J plugin. Two selected faces of the 3D rendering were then thresholded and used to count ER–mitochondria contact sites. Mitochondrial morphology: cells expressing the 4mt-circular permuted Venus (cpV) were morphologically analyzed, as described previously [28], using the Fiji software.

### 2.11. Ca^2+^ Experiments

Aequorin experiments were carried out on a PerkinElmer EnVision plate reader equipped with a two-injector unit. Cells were seeded in 24-well plates and then re-plated into 96-well plates (1:5 dilution) the day before the experiment. After reconstitution with 5 μM coelenterazine, cells were placed in 70 μL of extracellular saline (in mM: 140 NaCl, 2.8 KCl, 2 MgCl_2_, 10 HEPES, 1 CaCl_2_, 5 glucose; pH 7.4 at 37 °C) and luminescence from each well was measured for 1 min. During the experiment, 100 µM ATP was first injected at the desired concentration to activate Ca^2+^ transients in 0.5 mM EGTA-containing extracellular saline in the absence of Ca^2+^, and then a hypotonic, Ca^2+^-rich, digitonin-containing solution (10 mM CaCl_2_ in H_2_O) was added to discharge the remaining aequorin pool. Output data were analyzed and calibrated with a custom-made macro-enabled Excel workbook.

### 2.12. Real-Time Polymerase Chain Reaction

Total RNA was purified using a Quick RNA Miniprep Plus kit (Zymo Research) according to the manufacturer’s instructions and reverse transcribed using the SensiFAST cDNA Synthesis KIT (Bioline, Aurogene). Real-time polymerase chain reaction (RT-PCR) was performed using SensiFAST SYBR (Bioline). Primers were designed using PRIMER 3 software and purchased from TIB MOLBIOL (Genoa, Italy). Primer sequences and RT-PCR conditions are available upon request.

The following gene levels were analyzed: isoforms 2 and 3 of sarco/endoplasmic reticulum Ca^2+^-ATPase (SERCA), ryanodine receptors (RYR), and isoforms 2 and 3 of inositol 1,4,5-trisphosphate receptors (IP3R). Target gene levels were normalized to that of glyceraldehyde-3-Phosphate dehydrogenase (GAPDH) and hypoxanthine–guanine phosophoribosyltransferase (HPRT) mRNA. The Q-Gene program was used for analyzing gene expression [29].

### 2.13. Statistical Analysis

All experimental groups were studied in *n* ≥ 3. Data are presented as mean  ±  standard deviation (SD). Differences among the experimental conditions were tested using analysis of variance (ANOVA), as appropriate. Statistical significance was considered for *p* values  <  0.05. Statistical analyses were performed using SPSS software (Version 26, IBM).

## 3. Results

### 3.1. Proliferation Rate

**Figure 1:**
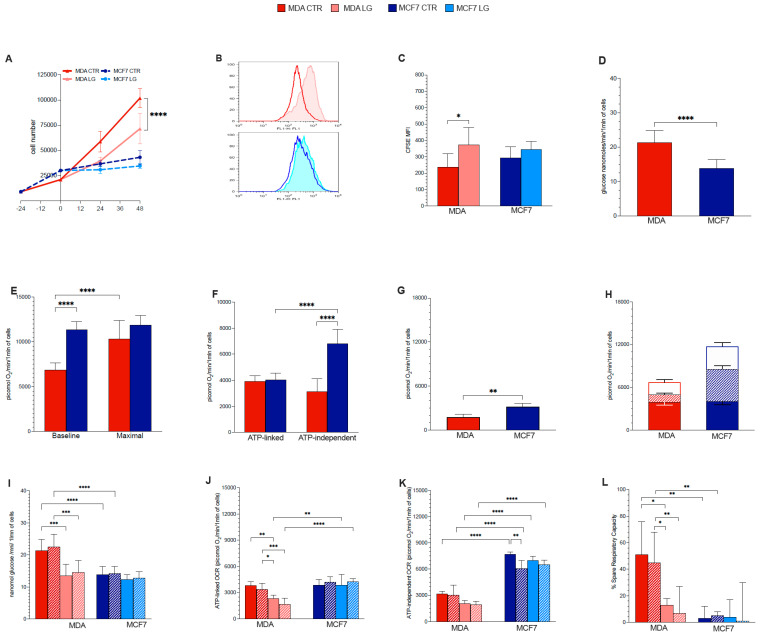
Glutamine effect on proliferation rate and metabolic profiles of breast cancer cells. (**A**) Growth curves of MDA (red solid lines) and MCF7 (blue dashed lines) cells cultures under control (CTR, dark lines) or partial glutamine deprivation (LG, pale lines). Number of independent experiments *n* ≥ 3. (**B**) Representative CFSE intensity histograms of MDA (red) and MCF7 (blue) cells proliferation after 48 h culture in CTR (dark) or LG (pale) medium. (**C**) Bar graphs displayed CFSE mean fluorescence intensity (MFI) of both MDA (red columns) and MCF7 (blue columns) cell types in the two conditions CTR (dark columns) and LG (pale columns). *n* ≥ 4. (**D**) Glucose consumption through glycolysis in MDA (red column) and MCF7 (blue column). (**E**) Oxygen consumption rate (OCR) under CTR condition (Baseline) and after mitochondrial uncoupling by FCCP (Maximal). (**F**) ATP-linked and ATP-independent OCR in MDA and MCF7 cells under CTR condition (*n* ≥ 15). (**G**) Extra-mitochondrial OCR measured (after rotenone/antimycin A administration) under CTR condition in both cell lines. *n* ≥ 3. (**H**) Summary of oxygen usage fractions: ATP-linked (solid columns), mitochondrial OCR-ATP independent (dashed columns) and extra-mitochondrial OCR (white columns) in MDA and MCF7 cells under CTR condition. *n* ≥ 3. (**I**) Glucose consumption through glycolysis under CTR conditions (dark solid columns), under LG (pale solid columns) and after BPTES administration in CTR (dark dashed columns) and LG (pale dashed columns) cultures (*n* ≥ 3). (**J**) ATP-linked OCR in the different cell lines and experimental conditions, coded as in panel I (*n* ≥ 3). (**K**) ATP-independent OCR in the different cell lines and experimental conditions, coded as in panel I (*n* ≥ 3). (**L**) % of spare respiratory capacity (measured as the difference between maximal and baseline OCR divided baseline OCR) n the different cell lines and experimental conditions, coded as in panel I. n ≥ 3. Data are represented as mean ± SD. * = *p* < 0.05; ** = *p* < 0.01; *** = *p* < 0.001; **** = *p* < 0.0001.

Prolonged (48 h) glutamine shortage significantly decelerated the growth curve of the aggressive triple-negative MDA cell cultures, while it had only a modest and not significant effect on MCF7 ones (Figure 1A). This response was not related to an increased mortality rate, since propidium iodide negative cells remained stable under control conditions and during nutrient stress both in MDA cells (88 ± 4.2 vs. 87 ± 3.1 %, respectively, *p* = ns) and in MCF7 ones (76 ± 3.9 vs. 71 ± 5.8%, respectively, *p* = ns). By contrast, it was related to a deceleration of proliferating activity as documented by the dilution curve of CFSE that was significantly altered only in the triple negative cancer model (Figure 1B,C).

### 3.2. Warburg Effect Is Independent of Energy Needs

In agreement with previous studies [30,31], culture growth and proliferation rate of MDA cells were dependent upon high levels of glutamine that, instead, was scarcely effective on MCF7 ones. To verify whether this addiction involved energy-related metabolism, we estimated OCR and lactate production, as markers of respiratory activity and glucose consumption through glycolysis (MRGlu).

MDA cells showed an evident Warburg effect leading to MRGlu values 54% higher with respect to MCF7 ones (*p* < 0.001, Figure 1D). By contrast, OCR showed the opposite pattern, being 60% higher in the latter with respect to the former ones (*p* < 0.001, Figure 1E). Despite this disparity and in agreement with previous reports [32], FCCP-induced mitochondrial uncoupling smoothed this difference and selectively increased OCR only in MDA cells, while being virtually ineffective in MCF7 ones (Figure 1E). Similarly, the difference in O_2_ usage was not dependent on energy metabolism. Indeed, ATP synthase-dependent OCR was remarkably similar in both cell lines (Figure 1F), while the ATP-independent oxygen consumption was markedly lower in MDA with respect to MCF7 cells (Figure 1F). Similarly, the administration of rotenone and antimycin A showed that the nonmitochondrial OCR was markedly lower (about twofold) in MDA with respect to MCF7 cells (Figure 1G).

Thus, the whole picture, represented in Figure 1H, indicated that the ATP-linked OCR accounted for 59% of total O_2_ usage in MDA cells, and only 34% in MCF7 ones. Moreover, the high energy-independent OCR of hormone-sensitive cells involved both mitochondria and non-mitochondrial structures. In other words, these data indicate that the high glycolytic rate configuring the so-called “Warburg effect” is scarcely related to energy production.

### 3.3. Glutamine Availability and Non-Respiratory Mitochondrial Function

Previous analyses documented that the high proliferation rate of MDA cells is dependent upon a high glutamine availability. Surprisingly, however, the activated Warburg effect of these same cultures was independent of OXPHOS activity, and rather reflected a low rate of energy-independent and extramitochondrial OCR.

Together with the behavior of proliferation rate, the activation of the Warburg effect in MDA cells seemingly suggested a relatively low relevance of glutamate usage in the TCA cycle. Accordingly, we aimed to verify the response of glycolysis and OCR to glutaminase inhibition by BPTES, to glutamine shortage (from 2 to 0.5 mM for 48 h), or their combination. MRGlu of MDA cells was largely decreased by glutamine deprivation while being insensitive to BPTES, regardless of the nutrient composition of the culture medium (Figure 1I). By contrast, lactate release of MCF7 cultures was fully independent of both glutaminase inhibition and amino acid shortage (Figure 1I).

In agreement with the MRGlu response, lowering glutamine availability to the physiological concentration also decreased the ATP-related OCR fraction (Figure 1J), while scarcely affecting the oligomycin-insensitive one (Figure 1K). On the other hand, glutaminase inhibition by BPTES was virtually ineffective regardless of the amino acid content of the culture medium (Figure 1J,K).

The combined decrease of MRGlu and OCR in MDA cells might indicate an indirect response of energy-producing processes to an impairment of glutamine-dependent anabolic pathways. Nevertheless, a direct effect of glutamine on mitochondrial function was reported by the analysis of uncoupled OCR. Indeed, both BPTES and glutamine shortage virtually abolished the response to FCCP (also called spare respiratory capacity) of the triple-negative cancer cells. More importantly, this effect was largely additive (synergism analysis, *p* = 0.868) (Figure 1L), despite the acknowledged notion that both stressors eventually interfere with glutamate generation. By contrast, the metabolic pattern of MCF7 cultures was virtually insensitive to either stressor or their combination (Figure 1L). Similarly, the already absent response to FCCP under control conditions was left unaltered by either stress.

### 3.4. Permissive Role for Glutamine in NADH Oxidation and NADP Reduction in Cancer Cell

These functional evaluations of the metabolic phenotype thus suggested a strict link between the Warburg effect and glutamine addiction, at least in MDA cultures. The observed deceleration in the proliferation rate of these “glycolytic cells” apparently agrees with the documented pivotal role of glutamine in feeding the anabolic processes [33]. However, the glutaminase-independent impairment in the maximal respiratory capacity paralleled the relative insensitivity of the highly oxidative MCF7 cells to glutamine abundance. Accordingly, this response might reflect a primary impairment of energy-producing pathways whose main intermediates were thus assayed using a metabolomic approach.

**Figure 2:**
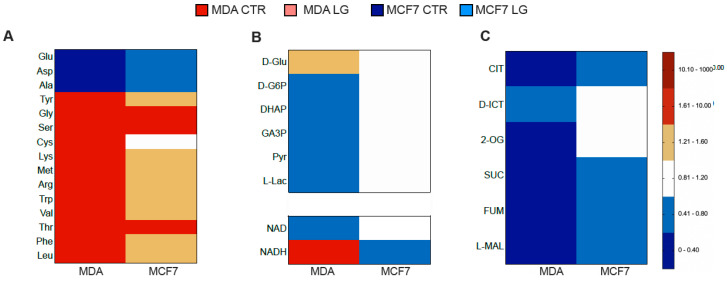

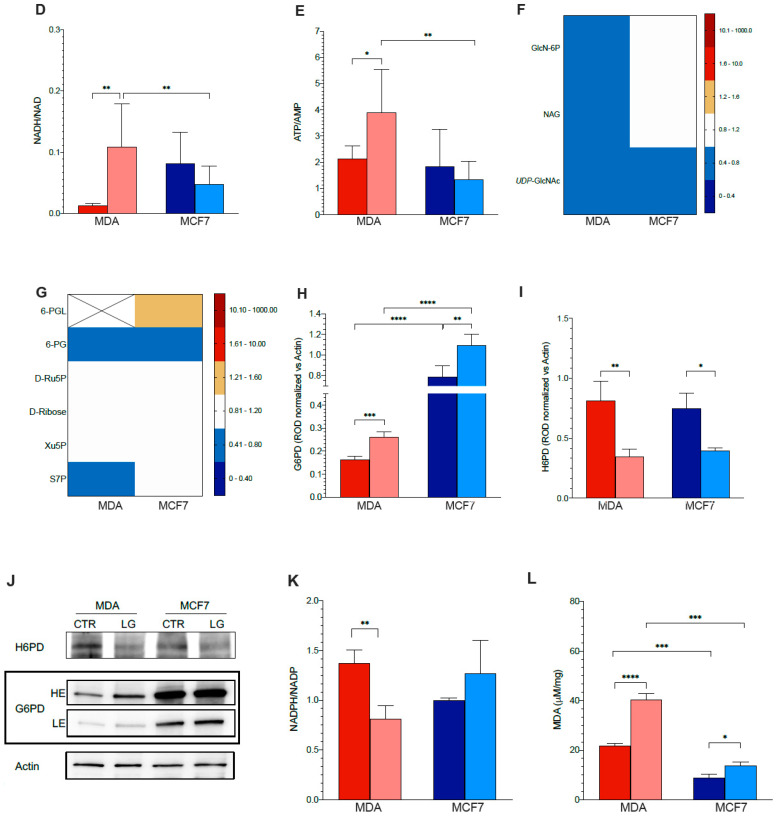
Metabolomic profiling of partial glutamine deprivation regulated pathways. (**A**–**C**) Metabolomic analysis in MDA and MCF7 cells: heat maps representation of substrate levels response to low glutamine condition (LG) by free amino acids, glycolysis intermediates and TCA metabolites, respectively (*n* ≥ 6). (**D**) NADH/NAD ratio (*n* = 7) and (**E**) ATP/AMP ratio of MDA (red) and MCF7 (blue) cultures under CTR (dark) and LG (pale) condition (*n* = 8). Heat maps representation of glutamine shortage effect on substrate levels of glucosamine pathway (**F**) and PPP (**G**) in MDA and MCF7 cells. (**H**,**I**) Relative optical density (ROD), normalized versus the housekeeping signal, of G6PD (**H**) and H6PD (**I**) in MDA (red) and MCF7 (blue) cells grown under CTR (dark) or LG (pale) condition, *n* = 3. (**J**) Representative WB signals of G6PD, H6PD and β-Actin, used as the housekeeping protein: HE and LE represent high and low exposure picture, respectively. (**K**) NADPH/NADP ratio and (**L**) quantification of malondialdehyde (MDA) content in cells grown under CTR or LG condition *n* = 3. Data are expressed as mean ± SD. Data are represented as mean ± SD. * = *p* < 0.05; ** = *p* < 0.01; *** = *p* < 0.001; **** = *p* < 0.0001.

The primary inhibition induced by glutamine shortage on protein synthesis was confirmed by the decreased intracellular content of all free amino acids but those directly generated by glutamine processing (aspartate, alanine, and glutamate). This response occurred in both cell lines, although markedly more pronounced in MDA than in MCF7 cells (Figure 2A).

By contrast, the nutrient stress dropped all glycolysis intermediates downstream of free glucose only in MDA cells (Figure 2B). Together with the absent response of MCF7 cultures, this metabolomic picture reproduced the previously reported functional evaluation. However, the slowdown in glycolytic flux was associated with a seven-fold increase of intracellular NADH, confirming the expected impairment of its exchanges between cytosol and mitochondrion through to the dedicated shuttles (Figure 2B). Indeed, the decreased content of all constituents of both aspartate–malate and glycerol-3-phosphate–glutamate shuttles further aggravated the shrinkage of all TCA metabolites, particularly in MDA cells (Figure 2A,C). However, this explanation does not fit several considerations. Indeed, the NADH excess should have been associated with high levels of glyceraldehyde-3-phosphate, whose content was instead decreased (Figure 2B), suggesting a preserved rate of its degradation by NAD^+^-dependent triose-phosphate dehydrogenase catalytic function. Likewise, cell lactate concentration (Figure 2B) and release (Figure 1K) were decreased by glutamine shortage despite the opposite expectation predicted by a high NADH/NAD^+^ ratio (Figure 2D). Finally, ATP/AMP ratio was markedly and selectively increased in MDA cells (Figure 2E). Accordingly, we speculated that the main response to glutamine shortage was characterized by decreased energy usage followed by a deceleration of energy-producing pathways, at least in the selected model of triple-negative breast cancer.

In addition to the impairment of anabolic processes, the deceleration of glycolytic flux might have been caused by an increased abundance of UDP-N-Ac-Gla that directly inhibits G6P channeling to glycolysis by the catalytic function of phospho-fructo-kinase-1 [12]. Nevertheless (and expectedly) this signaling molecule was markedly decreased in both cell lines although its precursors glucosamine-6P and N-Acetyl-D-glucosamine were only decreased in MDA cells (Figure 2F). Accordingly, the decreased glycolytic flux was not caused by the interference of the hexosamine biosynthetic pathway.

A similar observation also involved the G6P flux through the PPP. Although the levels of its intermediates were close to the detection threshold [34], glutamine shortage significantly decreased 6P-gluconate, i.e., the product of the PPP rate-limiting step. This response indicates a decelerated G6P flux through this pathway, particularly in MDA cells, as confirmed by the evident drop in sedoheptulose-7P [35] (Figure 2G). Yet, this same observation does not agree with the response of 6P-gluconolactone, which was undetectable in MDA cells, while it was significantly increased by partial glutamine deprivation in MCF7 ones (Figure 2G).

6P-gluconolactone is the direct, labile product of the reaction catalyzed by G6PD. By contrast, its reticular counterpart H6PD catalyzes the first two PPP reactions directly degrading G6P to 6P-gluconate [14]. Accordingly, the divergent trend of 6P-gluconolactone might indicate a different response to glutamine shortage by the ER- and cytosolic PPP.

To verify this hypothesis, we first tested the protein abundance of both enzymes. WB analysis (Figure 2H–J) documented a lower abundance of G6PD in MDA than in MCF7 cells, while H6PD levels were similar in both cultures. Glutamine shortage was followed by a marked enhancement of G6PD levels (Figure 2H,J), as opposed to a drop in H6PD expression (Figure 2I,J), in both cell types. In addition to agreeing with the PPP deceleration suggested by the decrease of sedoheptulose-7P, H6PD depletion was also associated with a decreased NADPH/NADP ratio again in MDA cells (Figure 2K). This imbalance was associated with evident redox damage, as documented by the significant increase in malondialdehyde concentration, as an index of lipid peroxidation, in both cell lines (Figure 2L).

### 3.5. Reticular PPP as a Selective Target of Glutamine Shortage

Overall, the full evaluation of intermediates and cofactors of glycolysis documented that glutamine shortage was associated with decreased glycolytic flux and lactate release despite the preservation of glucose availability, the excess in NADH-reductive power and the loss of the inhibitory effect of UDP-GlcNAc on phospho-fructokinase. It was also associated with a decreased glucose flux through the PPP that surprisingly disagreed with the behavior of G6PD expression as documented by the appearance of measurable levels of 6P-gluconolactone. By contrast, the decreased NADPH/NADP ratio fitted the behavior of H6PD in MDA cells but not in MCF7 ones. This difference potentially configures the ER-PPP as a preferential target for glutamine shortage and a primary supplier of NADPH reductive power, at least in the selected model of aggressive, triple-negative breast cancer.

Nevertheless, the decreased expression of H6PD occurred in both cell lines despite the different behavior of the estimated PPP rate. Obviously, the level of this triggering enzyme is not the unique determinant of glucose flux through the reticular pathway. On the other hand, the superimposable sequence of reactions downstream G6PD and H6PD prevents ascertaining the selectivity of glutamine addiction of G6P flux through the ER-PPP in MDA cells. Nevertheless, previous studies by our and other groups [21,25,36], already documented that its “omnivore” nature configures H6PD as the main determinant for the accumulation of the tracer used in the clinical setting to evaluate cancer metabolism, i.e., FDG. This concept partially disagrees with the commonly accepted equivalence between the direct measurement of MRGlu and its estimation based on tracer uptake (MRGlu*). Nevertheless, this kinetic model has been reproduced in a broad panel of cancer and normal cell lines both in vitro and in vivo and, so far, provides the most reliable explanation for the variability in MRGlu/MRGlu* ratio, called lumped constant, that has been described in a large literature body [37,38,39,40]. Indeed, H6PD gene silencing [25] or pharmacological inhibition of its catalytic function [21,41] strikingly decreased tracer retention, despite a marked increase in glycolytic flux.

We thus evaluated whether the decreased expression of H6PD and the decreased NADPH/NADP ratio induced by glutamine shortage were matched by an altered FDG accumulation kinetics as an index of ER-PPP function in both cell lines.

**Figure 3:**
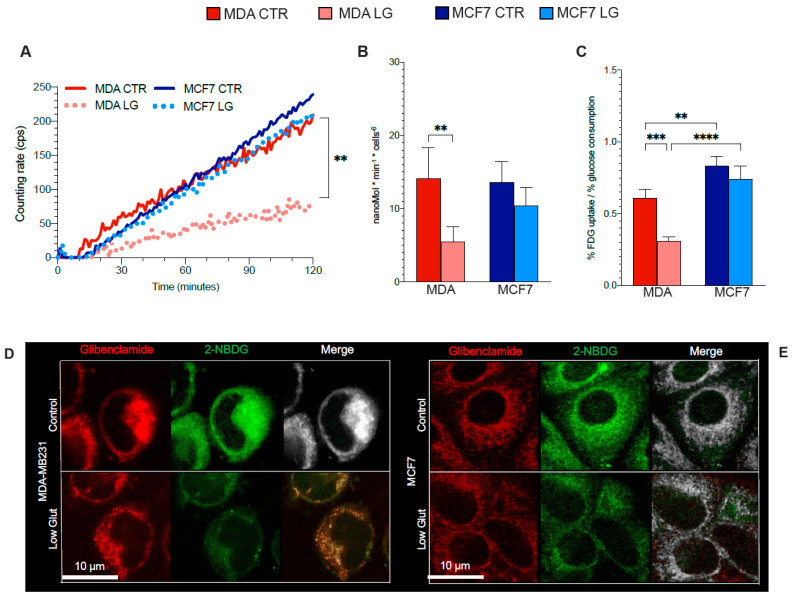

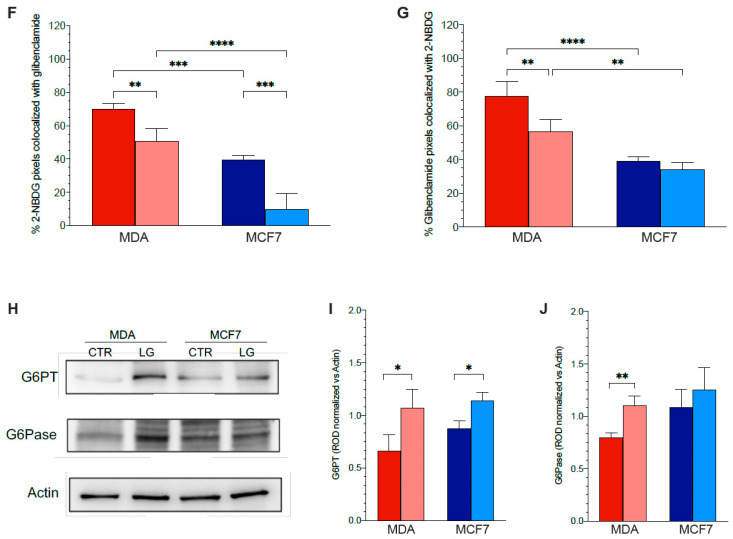
Glutamine availability affects G6P accumulation within the endoplasmic reticulum. (**A**) Time-activity curve of FDG kinetic uptake in MDA (red) and MCF7 (blue) cells grown under control (solid line) or partial glutamine deprivation (dashed line). *n* ≥ 4. (**B**) Metabolic rate of FDG (MRGlu*) calculated applying the conventional Sokoloff model of MDA (red columns) cells and MCF7 (blue columns) ones grown under control (CTR, dark columns) or partial glutamine deprivation (LG, pale columns) condition. *n* ≥ 4. (**C**) Experimental lumped constant (LC) value, representing the ratio between the % FDG uptake (evaluating by LigandTracer) and % glucose consumption (evaluating by Seahorse) of the two cell lines and under the two experimental conditions. *n* = 4 (**D**,**E**) Representative confocal microscopy images of glibenclamide (red), 2-NBDG (green) and merged (white) fluorescence signals in MDA cells (**D**) and MCF7 ones (**E**) grown under CTR or LG condition. (**F**,**G**) Analysis of signal colocalization expressed as percent of 2NBDG-containing pixels that colocalize with glibenclamide-positive ones (**F**) and glibenclamide-containing pixels that colocalize with 2NBDG-positive ones (**G**), for the two cell types in the two experimental conditions. *n* ≥ 3. (**H**) Representative western blot Scheme 6. phosphate transporter (G6PT), glucose-6-phosphatase (G6Pase), and β-Actin, used as the housekeeping protein. Densitometric analysis of WB signals normalized versus the housekeeping signal of (**I**) G6PT and (**J**) G6Pase of MDA cells and MCF7 ones grown under CTR or LG condition. Data are reported as % of respective control. *n* = 3. Data are represented as mean ± SD. * = *p* < 0.05; ** = *p* < 0.01; *** = *p* < 0.001; **** = *p* < 0.0001.

The FDG-derived index of glucose consumption MRGlu* was markedly decreased in glutamine-deprived MDA cells (Figure 3A,B) to values far below the corresponding response of MRGlu, directly measured by the Seahorse approach in the twin cultures (Figure 1J). By contrast, MCF7 cells showed a comparable response of direct and tracer-based estimation of glucose consumption (Figure 3A,B). Overall, the nutrient stress markedly and selectively decreased the lumped constant value in the triple-negative breast cancer model (Figure 3C).

To further verify the ER-PPP impairment, we evaluated whether the glutamine shortage actually hampers the accumulation of the fluorescent FDG analog 2-NBDG within the ER (Figure 3D,E). At confocal microscopy, the intracellular distribution of 2-NBDG was impaired by glutamine deprivation that, as reported in Figure 3D–G, decreased the colocalization of its signal with the corresponding index provided by the reticular probe glibenclamide.

We thus hypothesized that the mismatch between the responses of H6PD abundance and ER-PPP activity might have been caused by a divergent behavior of the two known determinants of G6P availability within the ER. On one side, the access of G6P and FDG-6P to the ER lumen asks for a transmembrane transport that is granted by the ATP-dependent G6P transporter (G6PT). On the other side, H6PD competes with G6P-phosphatase (G6Pase), which catalyzes the hydrolysis of phosphorylated hexoses and their escape from the ER. WB analysis (Figure 3H) showed that glutamine shortage increased G6PT abundance in both cell lines (Figure 3I), while the G6Pase level was selectively augmented in MDA cells (Figure 3J). Thus, the deceleration of glucose flux through the ER-PPP of MDA cells was explained by the combination of an impaired input of G6P (due to the downregulation of H6PD expression), and an enhanced glucose efflux due to the high dephosphorylation rate catalyzed by increased levels of G6Pase.

### 3.6. ER–Mitochondria Connection is Dependent upon ER-PPP Activity

A large body of literature has already documented a fundamental role for ER–mitochondria juxtapositions in regulating different functions of these two organelles, such as lipid biosynthesis, Ca^2+^ transfer, mitochondrial dynamics, autophagy, apoptosis, and inflammation [42]. Likewise, we previously reported that H6PD activity is needed to warrant this reticular function [25,34]. Thus, the decreased maximal OCR might reflect the consequence of the ER-PPP impairment induced by glutamine shortage.

**Figure 4:**
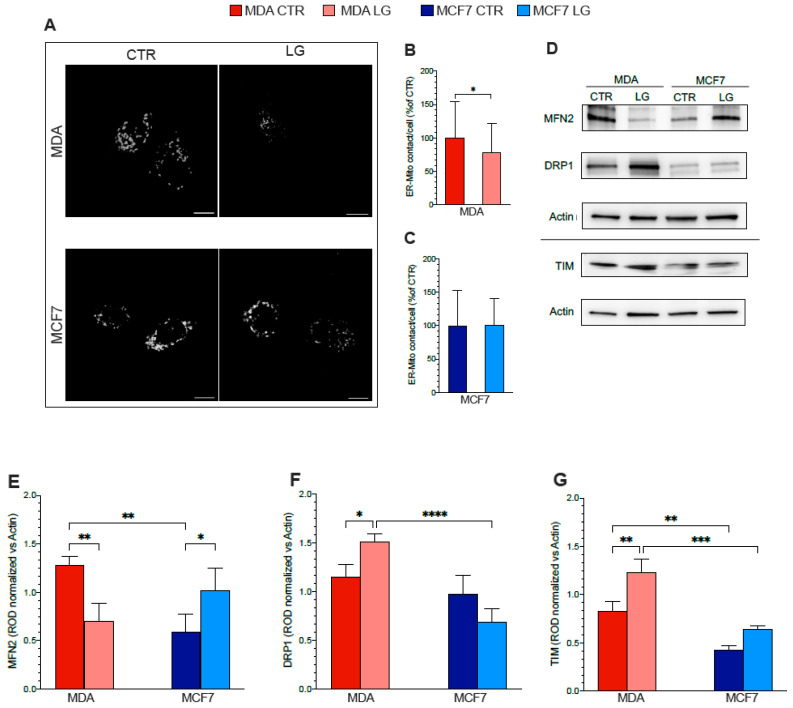

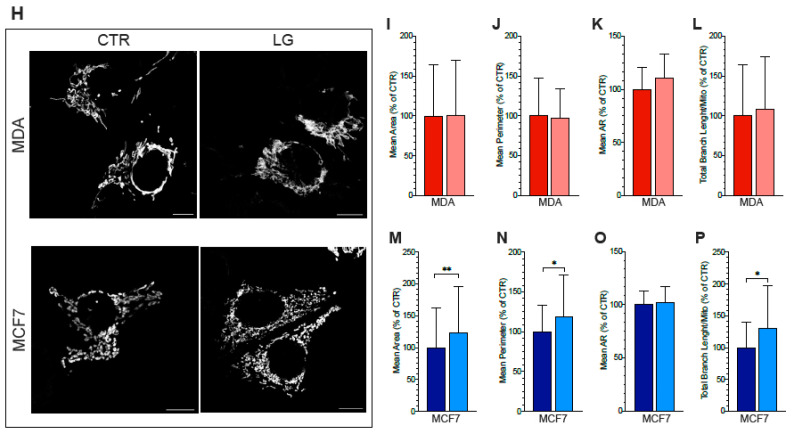
Glutamine availability and ER-Mitochondria relationship. (**A**) Representative confocal images of MDA and MCF7 cells grown in control (CTR) or in low glutamine (LG) conditions and expressing the SPLICSs probe. Scale bar 10 μm. (**B**) ER-mitochondrial contact points expressed as mean number of dots ± SEM obtained in (**B**) MDA cells and (**C**) MCF7 ones, grown under CTR (dark columns) or LG (pale columns) condition. *n* ≥ 39 cells of 4 independent transfections. (**D**) Representative western blot signals of MAMs protein and mitochondrial structure and β-Actin, used as the housekeeping protein. (**E–H**) Densitometric analysis of WB signals normalized versus the housekeeping signal of (**E**) MFN2, (**F**) DRP1, (**G**) TIM. Data are reported as % of respective control. *n* = 3. (H) Representative confocal images of MDA and MCF7 cells grown in CTR (left) or LG (right) condition and expressing the 4mt-cpV. Scale bar 10 μm. (**I**–**P**) Mitochondrial morphology and network analysis in MDA cells and MCF7 ones grown in normal CTR (dark columns) or in LG (pale columns) medium. (**I**,**M**) Mitochondrial area and (**J**,**N**) perimeter of MDA (**I**,**J**) and MCF7 (**M**,**N**) cells cultured under CTR or LG condition. (**K**,**O**) Aspect ratio and (**L**,**P**) total length of branches MDA (**K**,**L**) and MCF7 (**O**,**P**) cells grown in CTR or LG medium. *n* = 41 cells for MDA and *n* = 38 cells for MCF7 of 4 independent transfections. Data are represented as mean ± SD. * = *p* < 0.05; ** = *p* < 0.01; *** = *p* < 0.001; **** = *p* < 0.0001.

To test this hypothesis, we verified whether the nutrient stress affects the ER–mitochondria proximity differently using the SPLICS in the two cell types (Figure 4A). The relatively lower impairment of ER-PPP actually matched an invariant organelles interaction in MCF7 cells (Figure 4A,C). By contrast, glutamine shortage induced a significant decrease (25%) in contact points of MDA cells (Figure 4A,B).

The observed alterations in ER–mitochondrial networking suggested the occurrence of an impairment in the fusion/fission machinery induced by amino acid deprivation. We thus performed WB experiments to test proteins involved in mitochondria dynamics and mass (Figure 4D). Among the factors contributing to mitochondrial fusion, Mfn2 was selectively decreased in MDA cells as opposed to MCF7 ones (Figure 4E). This observation matched the opposite behavior of Drp1, a GTPase involved in mitochondrial fission (Figure 4F), suggesting a selective enhancement of mitochondrial fission in MDA cells.

We thus verified whether the shift in molecular signaling was associated with a corresponding alteration to organelle morphology using 2D analysis expressing the mitochondrial-targeted, circularly permuted Venus (cpV) in fixed cells (Figure 4H). After prolonged glutamine deprivation, MCF7 cells showed a significant increase in the mitochondrial mean area (Figure 4 M,N); data were in line with the augmented TIM expression (Figure 4G). This response did not imply any significant variation in the mitochondrial shape and was, rather, associated with an increase in branches and junctions, indicating a major complexity of the organelle network (Figure 4O,P). By contrast, glutamine deprivation did not modify the size, network complexity, or connectivity of MDA mitochondria (Figure 4I–L) in which, however, the increase in TIM expression was associated with a significant elongation (Figure 4G).

### 3.7. ER–Mitochondrial Ca^2+^ Exchanges

The role of H6PD in modulating ER–mitochondria interaction might affect the Ca^2+^ shuttling between these two organelles [17]. We thus tested the effect of glutamine shortage on cytosolic and mitochondrial Ca^2+^ transients and localization, upon ATP stimulation, in both cancer cultures.

**Figure 5:**
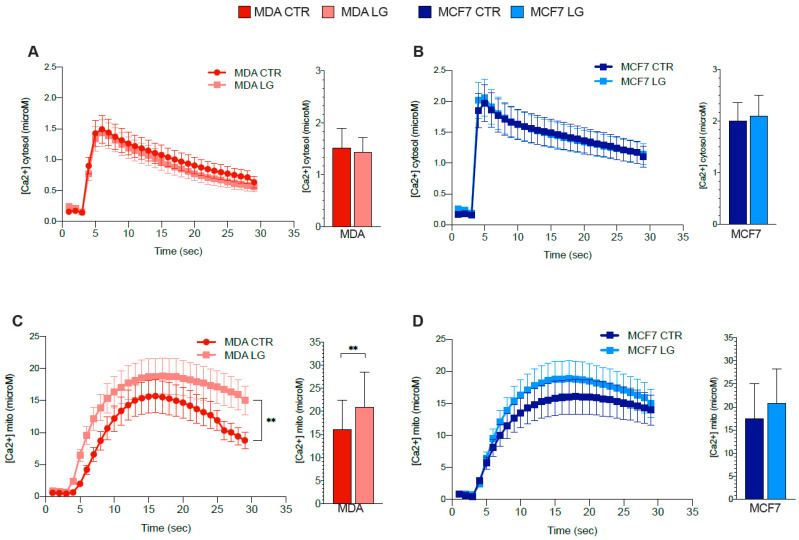

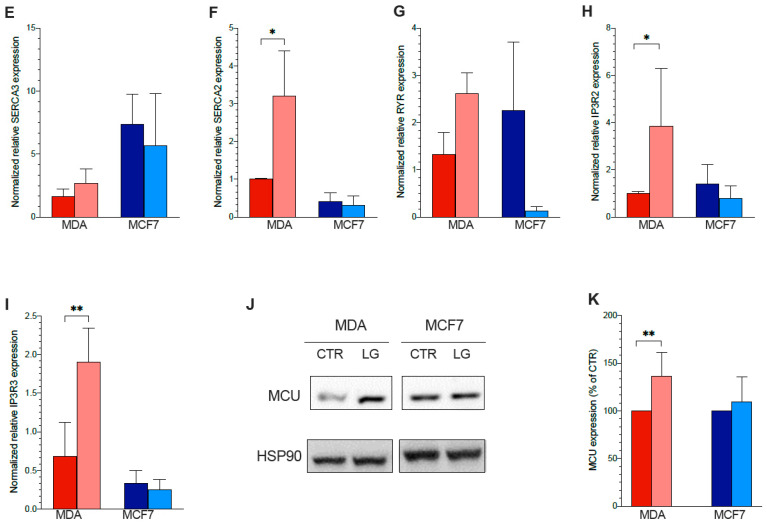
Glutamine availability and ER-mitochondrial Ca^2+^ exchanges. Average traces and peak amplitude of (**A**,**B**) cytosolic or (**C**,**D**) mitochondrial Ca^2+^ uptake in (**A**,**C**) MDA (red) or (**B**,**D**) MCF7 (blue) cells grown in control (CTR, dark) or in low glutamine (LG, pale) condition. *n* ≥ 35 cells of 4 independent transfections. Normalized mRNA expression of (**E**) SERCA3, (**F**) SERCA2, (**G**) RYR, (**H**) IP3R2 and (**I**) IP3R3 in MDA (red columns) or MCF7 (blue columns) cells cultured under CTR (dark columns) or LG (pale columns) condition. *n* = 3. (**J**,**K**) WB analysis of MCU and HSP90 (housekeeping protein) grown in CTR or LG culture medium. (**J**) Representative WB signals. (**K**) Densitometric analysis, reported as % of respective control. *n* = 6. Data are represented as mean ± SD. * = *p* < 0.05; ** = *p* < 0.01.

Cytosolic Ca^2+^ transients were not altered by the nutrient stress in either cell type (Figure 5A,B). Nevertheless, mitochondrial Ca^2+^ content was increased by glutamine shortage only in MDA cultures (Figure 5C,D) and unaffected in MCF7 cells. Among the main channels dedicated to the Ca^2+^ accumulation within the ER, cancer cells largely express SERCA isoforms 2 and 3 [43]. Glutamine shortage did not affect mRNA levels encoding for SERCA 3, while it increased SERCA 2 gene expression only in MDA cells (Figure 5E,F). This response was associated with a parallel slight but not significant increase in RYR expression, which promotes Ca^2+^ efflux from ER to cytosol (Figure 5G). Obtained data thus suggested a relative balance of cation exchanges between these two compartments. Nevertheless, the Ca^2+^ accumulation in glutamine-deprived MDA mitochondria agreed with an increased mRNA expression of two transporters deputed to this transfer from the ER (Figure 5H,I)—IP3R isoforms 2 and 3. This response was further aggravated by the amplification of the electrochemical gradient between the mitochondria-associated ER membrane (MAM) region and the intermembrane mitochondrial space. Indeed, MCU levels were not significantly different under control conditions between the two cell lines (Figure 5J). By contrast, glutamine shortage selectively increased MCU abundance only in the triple negative aggressive cancer model (Figure 5J,K).

## 4. Discussion

Most neoplastic lesions are known to facilitate the flux of G6P into the PPP, branching from glycolysis at the first committed step of glucose metabolism. This metabolic shift allows fueling the building blocks and the reductive power needed by high-rate anabolic processes and antioxidant responses. In addition to its role as the unique source of ribose-5P for ribonucleotides synthesis, the capability of PPP to preserve the intracellular NADPH pool [12,44,45,46,47] largely exceeds the contribution of glutamate dehydrogenase [48], malic enzyme 1 [49] and isocitrate dehydrogenases in the noncanonical TCA direction [50]. Classical assumptions consider PPP as a cytosolic glucose metabolism triggered by G6PD. Nevertheless, glutamine deprivation simultaneously decreased the proliferation rate of MDA cells, several PPP intermediates, and the NADPH/NADP ratio. Combined with the evident oxidative damage, these observations indicate a slowdown in PPP activity that mismatched the marked increase in G6PD expression, observed in our experiments in agreement with previous studies [13].

This apparent paradox is explained by the opposite behavior of the PPP branch confined within the ER that, although scarcely considered, has been found to provide a high contribution to the ribose production and NADPH generation of several types of cancer cells [16,34]. Indeed, the expression of its triggering enzyme H6PD was decreased after glutamine shortage in both hormone-sensitive and triple-negative cultures. However, MDA cells coupled this response with an increased expression of G6Pase, able to prevent G6P access to H6PD due to the shared reticular confinement.

The metabolic correlate of this enzymatic impairment under glutamine deprivation is corroborated by several observations. Indeed, the combination of an evident glutamine addiction with a relatively low G6PD abundance agrees with a high dependence of the rapidly proliferating MDA cells on the ER-PPP. Likewise, the consequence of H6PD downregulation on NADPH reductive power and the consequent redox damage was particularly pronounced in these triple cells. Finally, the same MDA cells showed higher rates of both FDG uptake and reticular accumulation of its fluorescent counterpart 2-NBDG with respect to their hormone-sensitive counterparts with both indexes particularly impaired by glutamine shortage. A large body of evidence previously documented that, once phosphorylated by hexokinases, these two glucose analogs cannot be channeled to glycolysis and cytosolic PPP, being false substrates for phosphoglucose isomerase and G6PD, respectively [51]. By contrast, they can be—and indeed are—processed by the “omnivore” H6PD enzyme, whose catalytic function prevents their hydrolysis by G6Pase and the subsequent glucose transporter (GLUT)-mediated release to the cytosol. Accordingly, the agreement between tracer kinetics, enzymatic asset, and intermediate levels configures the deceleration of G6P flux through the ER-PPP as a primary determinant of the NADPH reductive power in cancer.

According to our protocol, implying partial amino acid deprivation, glutamine starvation was particularly severe in the aggressive model of MDA cultures that are relatively depleted of glutamine synthase as opposed to the preserved enzyme expression of MCF7 ones [52]. In agreement with this experimental design, glutamine shortage did not affect the culture growth of these hormone-sensitive cells, suggesting a direct dependence of MDA anabolic pathways on the glutamine excess typical of the in vitro cultures. A similar consideration also applies to the behavior of MRGlu and OCR, whose slowdown in the triple-negative breast cancer cells was associated with an increased ATP/AMP ratio. This response suggests an adaptation of energy-producing pathways to a decreased metabolic demand. More importantly, it also confirms that the high glycolytic flux typical of the Warburg effect is largely independent of energy needs.

On the other hand, the impairment in maximal respiratory capacity induced by glutamine deprivation confirms that this amino acid contributes to the regulation of mitochondrial activity, at least in triple-negative cells. This role has most often been attributed to the capability of glutamine-derived glutamate to supply the Krebs cycle with the nitrogen and carbon equivalents needed for its anaplerotic function, threatened by the high citrate efflux to the cytosol for fatty acid synthesis. However, these same data document a relevant role for ER–mitochondria networking in this setting. Indeed, the decreased number of ER–mitochondria juxtapositions induced by glutamine shortage were paralleled by MCU upregulation, increased mitochondrial Ca^2+^ uptake, and oxidative stress as a preliminary step for the downregulation of the MDA proliferation rate [53]. This finding reproduces previous observations about the mandatory role of H6PD function in preserving the ER–mitochondria networking, the cellular redox state, and thus the mitochondrial Ca^2+^ content. Similarly, it fits with the high levels of glutamine transporters that have been found to characterize the MAMs [54]. Accordingly, these data seem to suggest that glutamine addiction of rapidly proliferating cancer cells might involve the ER-PPP as a powerful, so far unrecognized, regulator of cancer metabolic reprogramming through its capability to modulate the mitochondrial function.

## 5. Conclusions

In conclusion, the present study indicates that ER-PPP contributes to the glutamine dependence of anabolic processes at least in the selected model of aggressive triple-negative breast cancer. Its reticular localization configures this pathway as a fundamental regulator of signals triggering cell proliferation through the ER–mitochondria connection.

Although the underlying mechanisms cannot be defined, the opposite response of H6PD and G6PD expression to glutamine shortage indicates profound independence of these two geographically unconnected pathways. Likewise, the agreement of the ER-PPP rate with the proliferating activity configures this reticular metabolism as a primary determinant of cancer aggressiveness.

This observation might open future windows for the therapeutic targeting of cancer metabolism. So far, they provide an alternative and robust explanation of the high diagnostic and prognostic power of 18F-FDG uptake. This clinical description of cancer aggressiveness would be independent of glucose consumption, the most elementary feature of life, shared by all living cells. Rather, it would derive from the capability of this tracer to describe the activity of a specific ER metabolism strictly connected with cancer growth.

## Data Availability

All data are contained within the article.

## Supplementary Materials

The following supporting information can be downloaded at: https://www.mdpi.com/article/10.3390/antiox12010043/s1, Figure S1. Calibration of the LigandTracer measurement.
