# Supplementary figures and images for: Fundamental Role of Pentose Phosphate Pathway within the Endoplasmic Reticulum in Glutamine Addiction of Triple-Negative Breast Cancer Cells
Source: Antioxidants (Basel). 2022 Dec 26;12(1):43. doi: 10.3390/antiox12010043 (PMC9854646; doi:10.3390/antiox12010043)

# Ligand-Tracer calibration

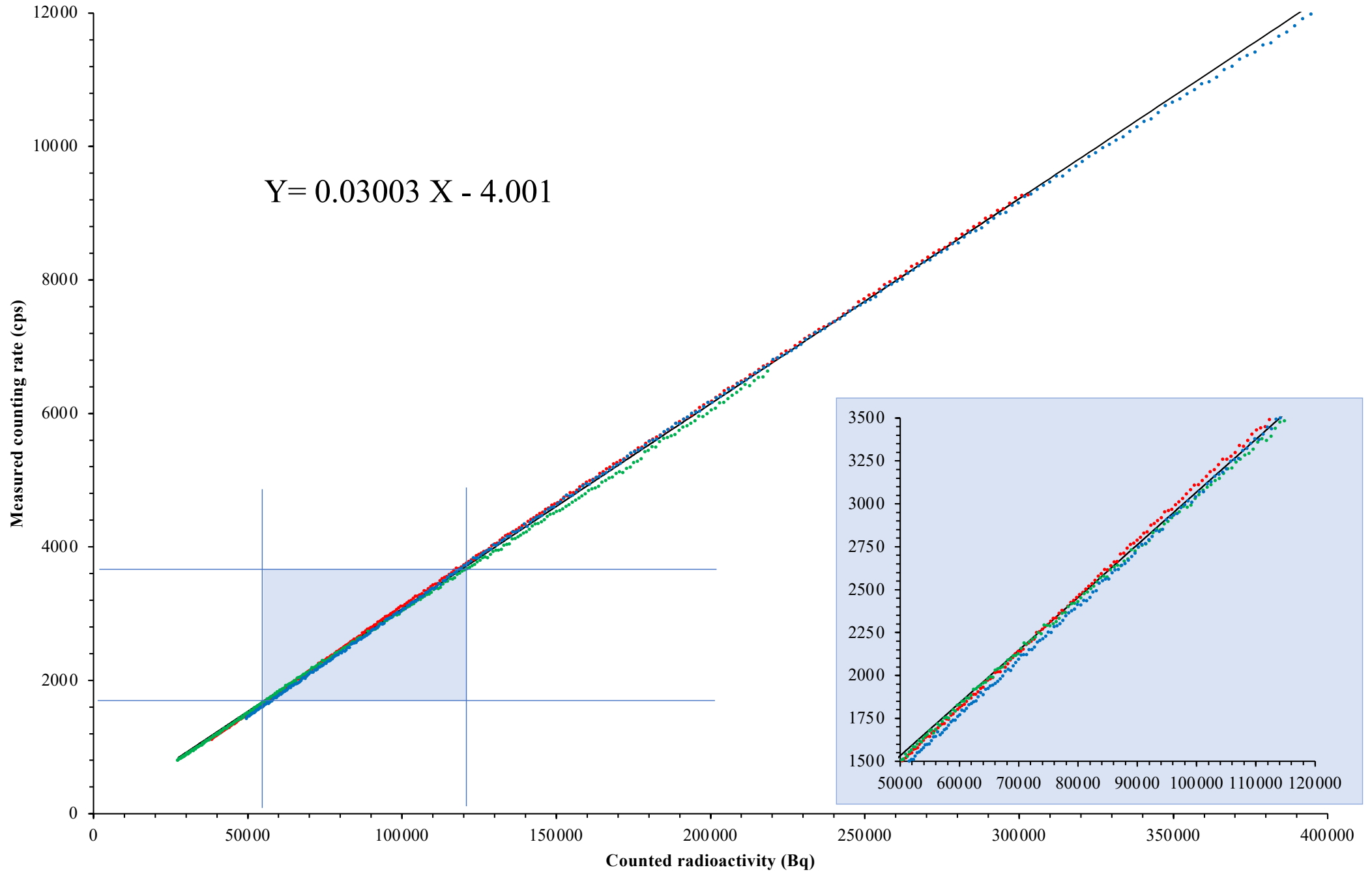

Supplement: Supplementary file 1 [file antioxidants-12-00043-s001.zip › antioxidants-2067439-supplementary.pdf]
